# Effectiveness of Platelet Rich Plasma and Bone Graft in the Treatment of Intrabony Defects: A Clinico-radiographic Study

**DOI:** 10.2174/1874210601812010133

**Published:** 2018-02-12

**Authors:** Mohammad Jalaluddin, Jayachandran Mahesh, Rethi Mahesh, Ipsita Jayanti, Mohamed Faizuddin, Krishna Kripal, Nazia Nazeer

**Affiliations:** 1 Department of Periodontics & Oral Implantology, Kalinga Institute of Dental Sciences, KIIT University, Bhubaneswar, Odisha, India; 2 Department of Periodontics, Noorul Islam College of Dental Sciences, Thiruvananthapuram, Kerala, India; 3 Department of Endodontics & Conservative Dentistry, Noorul Islam College of Dental Sciences, Thiruvananthapuram, Kerala, India; 4 Department of Periodontology and Oral Implantology, R.V.Dental College and Hospital, Bengaluru, Karnataka, India; 5 Department of Periodontology & Oral Implantology, Raja Rajeswari Dental College & Hospital, Bengaluru, Karnataka, India; 6 Kalinga Institute of Dental Sciences, KIIT University, Bhubaneswar, Odisha, India

**Keywords:** Bone graft, Infrabony defects, Periodontal regeneration, PRP. Platelet Derived growth factors (PDGF), Epithelial growth factor (EGF)

## Abstract

**Background & Objectives:**

Periodontal disease is characterized by the presence of gingival inflammation, periodontal pocket formation, loss of connective tissue attachment and alveolar bone around the affected tooth. Different modalities have been employed in the treatment and regeneration of periodontal defects which include the use of bone grafts, PRP and other growth factors.The purpose of this prospective, randomized controlled study was to compare the regenerative efficacy of PRP and bonegraft in intrabony periodontal defects.

**Methodology:**

This randomized control trial was carried out in the Department of Periodontics & Oral Implantology, Kalinga Institute of Dental Sciences and Hospital, KIIT University, Bhubaneswar. The study sample included 20 periodontal infrabony defects in 20 patients, 12 males and 8 females. The patients were aged between 25 -45 years(with mean age of 35 years). The 20 sites selected for the study were was randomly divided into 2 groups of 10 sites each. Group A**:** PRP alone, Group B**:** Bone Graft.

**Statistical Anaysis & Results: Statistical Analysis Was Done Using SPSS (Version 18.0):**

Statistical analysis was done usingpaired ‘t’ tests and ANOVA that revealed a significant reduction ingingival index, plaque index, probing pocket depth and gain in clinical attachment level at various time intervalswithin both the groups. Radiographic evaluation revealed statistically significant defect fill (*p*<0.001) at the end of 6months within both the groups. However, there was astatistically significant difference seen in group B radiographically, when compared to group A.

**Conclusion:**

Both the groups showed promising results in enhancing periodontal regeneration; however the resultswith bonegraftwere comparatively better, although not statistically significant when compared to PRP alone.

## 1 INTRODUCTION

Periodontal disease is one of the most prevalent afflictions worldwide and is the major cause of tooth morbidity and mortality. The disease is characterized by the presence of gingival inflammation, periodontal pocket formation, loss of connective tissue attachment and alveolar bone around the affected tooth. The regeneration and integration of multiple tissue types is critical for efforts to restore the function of musculoskeletal complex. In particular, the neogenesisof periodontal structures for systematic tooth supporting functions is a current challenge due to micron scale tissues compartmentalization (gingival fibers, periodontal fibers, alveolar bone, cementum) and the orchestration of multiple regenerated tissues [1]. The reconstruction or restorations of these defects caused by inflammatory periodontal disease is a continuing challenge in periodontal therapy. Although many attempts have been made to regenerate alveolar bone support and the attachment apparatus, predictable success has proved elusive [2]. This goal can be accomplished by using regenerative surgical procedure like root biomodification, use of bone replacement grafts, Guided tissue regeneration (GTR) and growth factors [2, 3]. There is evidence to suggest that present regenerative technique lead to significant amounts of regeneration at localized sites on specific teeth [4]. However if complete regeneration is to become a reality, additional stimuli to enhance the regenerative process are likely needed. Perhaps these could be attempted with polypeptide growth factors or biologic modifiers to provide additional stimulus. These factors may have the potential to promote regeneration through a variety of cell tissue interactions, including promoting cell migration, attachment and subsequent spreading of cells at the local site, cell proliferation, chemotaxis, differentiation and matrix synthesis [4].

Growth factors that seem to play an important role in periodontal and bone wound healing are platelet derived growth factors (PDGF) and transforming growth factor-β(TGF-β), Insulin like growth factor(IGF), Vascular endothelial growth factor(VEGF) and Epithelial growth factor(EGF) [5]. PDGF has been shown to exert a favorable effect on periodontal regeneration as measured by increase in clinical attachment levels and osseous defect fill in humans [6]. Topical application of TGF-β stimulates proliferation of gingival fibroblastic cells, formation of blood vessels and remodelling of extracellular matrix, which results in increased proliferation of granulation tissue within healing tissues [6]. TGF-β when coated onto β-Tricalcium phosphate pellets substantially stimulates cell proliferation and differentiation of osteoblast lineage cells and induces bone formation in rat calvarias osseous defects [7].

A convenient and economical approach to obtain autologous PDGF and TGF-β is the use of platelet rich plasma (PRP) [8]. PRP is obtained by sequestrating and concentrating platelets by gradient density centrifugation. The process of centrifugation concentrates human platelets 338% with identified PDGF and TGF-β within the concentrates [9, 10]. Delivery of autologous platelets to periodontal wounds can increase the local concentration of growth factors, which may enhance the healing outcomes.

Different types of bone grafts are used to fill the periodontal defects and restore the lost periodontal attachment apparatus. Alloplasts are synthetic, inorganic, biocompatible non-antigenic graft substitutes, which promote healing through osteoconduction [3]. No data is available for use of PRP alone in intrabony defects. It is yet unknown whether PRP alone may enhance the outcome of periodontal therapy. Therefore, the purpose of this prospective, randomized controlled study was to assess and compare the regenerative potential of PRP and bonegraft in intrabony periodontal defects.

## 2 AIMS & OBJECTIVES

The aim of this study was to evaluate the regenerative potential of Platelet Rich Plasma (PRP) in comparison tobonegraft on clinical parameters *i.e*., probing pocket depth, clinical attachment level, position of gingival margin and to assess radiographically the ability of PRP and bonegraft, to bring about bone fill in periodontal intrabony defects.

## 3 STUDY DESIGN & METHODOLOGY

This randomized controlled clinical trial was carried out in the Department of Periodontics & Oral Implantology, Kalinga Institute of Dental Sciences and Hospital, KIIT University, Bhubaneswar.

The study sample included 20 periodontal infrabony defects in 20 patients, 12 males and 8 females. The patients were aged between 25-45 years (mean age of 35 years). The defects were randomly divided into 2 groups using Coin flip test and followed up for a period of 6 months.

The 20 sites selected for the study were randomly divided into 2 groups of 10sites each.

**Group A:** PRP alone

**Group B:** Bone Graft

### 3.1 Patient Selection Criteria

#### 3.1.1 Inclusion Criteria

1. Patients with good general health without any history of systemic disease or compromising medical conditions.
2. Patients with clinical evidence of periodontal pocket with probing depths more than 5mm.
3. Intra bony defect.
4. Patients willing to cooperate and complete the duration of the study.

#### 3.1.2 Exclusion Criteria

1. Patients having unacceptable oral hygiene during presurgical phase (phase 1 therapy)
2. History of antibiotics or other medications affecting the periodontium, within the previous 6 months
3. Pregnant women and lactating mothers
4. mokers

### 3.2 Clinical Parameters Assessed

The following clinical parameters were assessed at baseline, three & six months after the surgical procedure. Radiographic bone level were assessed at the end of six months: Plaque index (PI), [11]. Gingival index (GI), [12]. Position of gingival margin (PGM), Probing pocket depth (PPD), Clinical attachment level (CAL), Radiographic bone level (RBL).

**Fig. (1):**
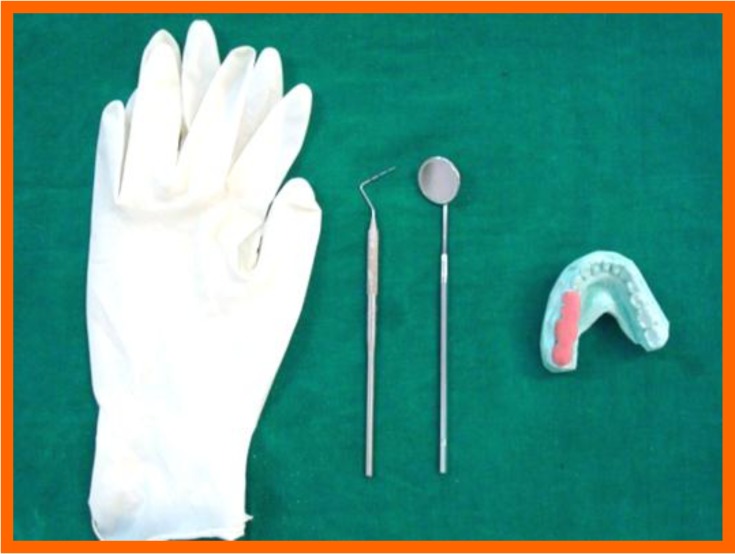
Clinical armamentarium.

Position of gingival margin, probing pocket depth and clinical attachment level were recorded by using a UNC-15 periodontal probe and a customized acrylic-stent with a guiding groove. This provided well defined and reproducible clinical measurements at each experimental and control site at baseline, 3 & 6 months. All the customized acrylic stent were stored on the prepared study casts throughout the study period to minimize distortion (Fig. **1**)

The following measurements were recorded [13].

1. The distance from reference point (RP) on the stent to the gingival margin (GM)
2. The distance from reference point on the stent to the base of the pocket (BOP)

Probing pocket depth (PPD) was recorded by noting the difference between measurements from reference point to gingival margin and reference point to the base of the pocket.

PPD=RP to BOP – RP to GM

Changes in the marginal gingival position were recorded by measuring the distance from fixed reference point to gingival margin.

### 3.3 Radiographic Assessment

1. Prior to surgery, intra oral periapical radiographs were taken using an Extension Cone Paralleling Device.
2. Kodak E speed films were used in a Siemens X-ray unit (70Kv, 15 ma, 0.6mas) and routine radiographs were taken. [14]. To standardize radiographic assessment, radiographs were obtained in a constant and reproducible plane using film holders with a template, which was placed in a constant position on a group of teeth and an extension arm that could be attached to the film as well as the X-ray tube (Fig. **1**).
3. Intra-oral periapical (IOPA) radiographs, were taken at baseline and at 6 months postoperatively.

### 3.4 Method for Radiographic Assessment

The following method was employed, to determine radiographic changes before and after the study [15].

#### 3.4.1 Pre-operative Measurement at Base Line

A - CEJ to base of defect (BOD)
B - CEJ to the Alveolar crest (AC)
C - Defect depth at base line (A-B)

#### 3.4.2 Post-Operative Measurements at 6 Months

A^1^ - CEJ to BOD
B^1^ - CEJ to AC
C^1^ - Defect depth at six months (A^1^ ± B^1^)
E - Changes in alveolar crest at six months (B ± B^1^)
D - Defect fill in mm (linear). (C ± C^1^)

### 3.5 Arithmetic Determinations

1. Defect depth in mm. C= A ± B C^1^ = A^1^ ± B^1^
2. Defect fill in mm. D = C ± C^1^ D^1^ = C ± C^2^
3. Changes in alveolar crest. E = B ± B^1^ E1 = B ± B^2^
4. Percentage of defect fill = [Defect depth at base line – Defect depth (6 months)] – Change in

$\frac{\mathrm{alveolar}\;\mathrm{crest}(6\mathrm{months}) \times 100}{\mathrm{Defect}\;\mathrm{depth}\;\mathrm{at}\;\mathrm{base}\;\mathrm{line}}$

1. Percentage of original defect resolution =

$\frac{\mathrm{Defect}\;\mathrm{depth}\;\mathrm{at}\;\mathrm{base}\;\mathrm{line}-\mathrm{Defect}\;\mathrm{depth}(6\;\mathrm{months}) \times 100}{\mathrm{Defect}\;\mathrm{depth}\;\mathrm{at}\;\mathrm{base}\;\mathrm{line}}$

**Fig. (2):**
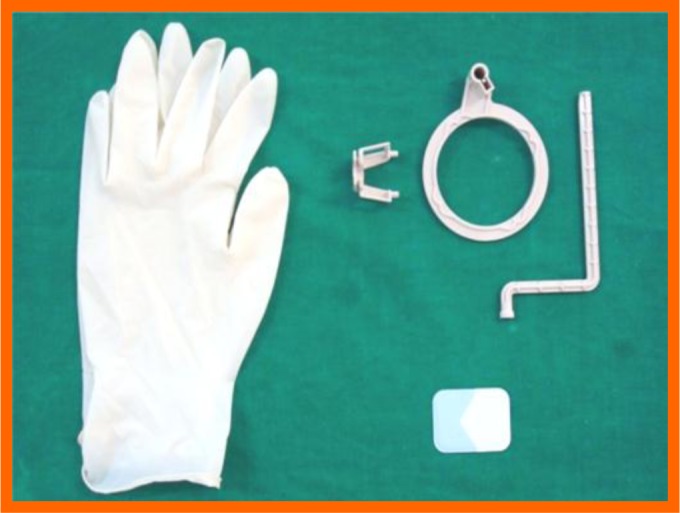
Radiographic armamentarium.

The radiographs were scanned on an hp x-ray scanner and measurements were carried out by the help of image analysis software (Auto-Cad) to see changes between preoperative and post-operative radiographs (Fig. **2**).

### 3.6 Bone Graft Material

**Fig. (3):**
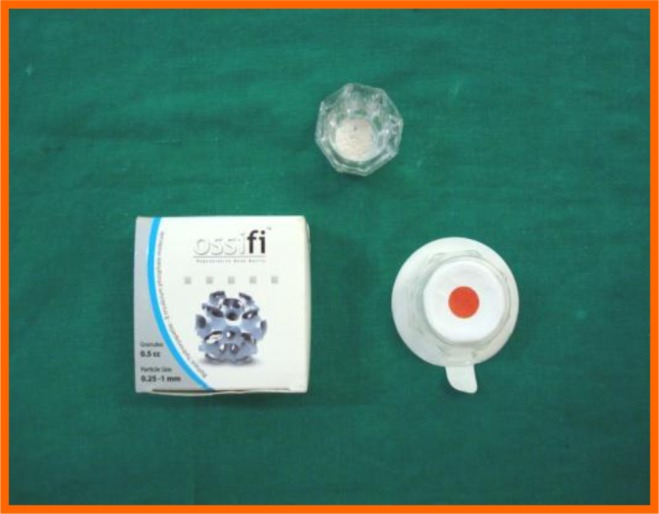
Bone graft.

Ossifi (Equinox), a biphasic hydroxyapatite-β-tricalcium phosphate molecule was used. It is a combination of hydroxyapatite and beta tricalcium phosphate in a 70/30 ratio which is extremely biocompatible and highly osteoconductive. The calcium phosphate composition enhances the biological resorption of the granule and ensures optimum bone ingrowth and formation. The 0.25-1 mm ossifi particle size is used in our study (Fig. **3**).

### 3.7 Preparation of Platelet Rich Plasma (PRP)

**Fig. (4):**
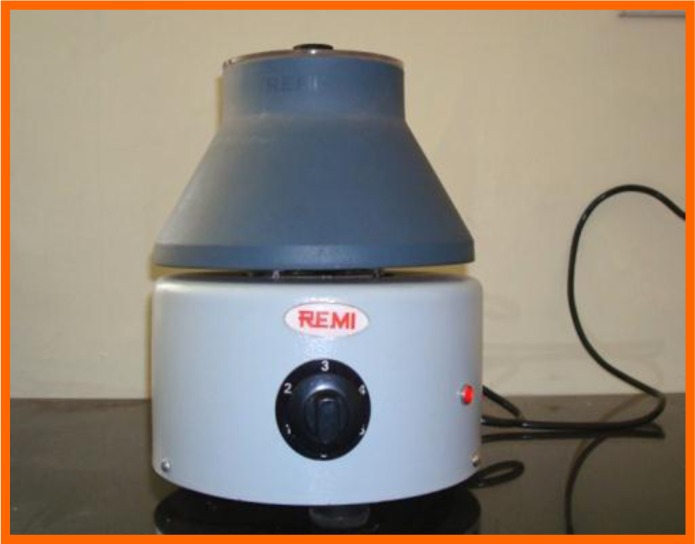
Centrifugal machine.

**Fig. (5):**
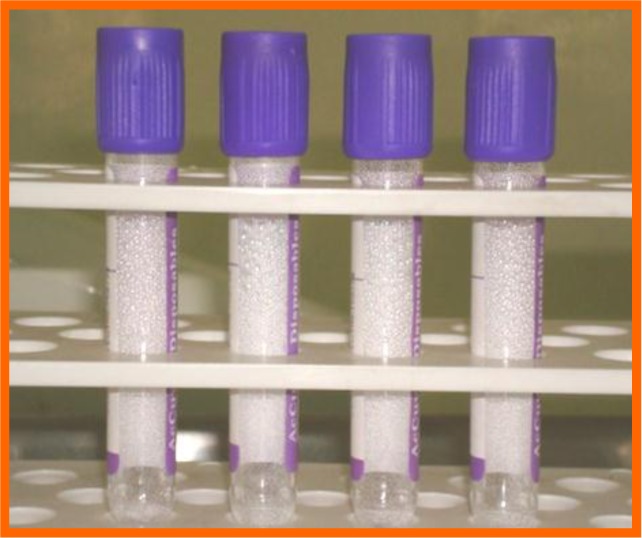
After 1^st^ centrifuge.

**Fig. (6):**
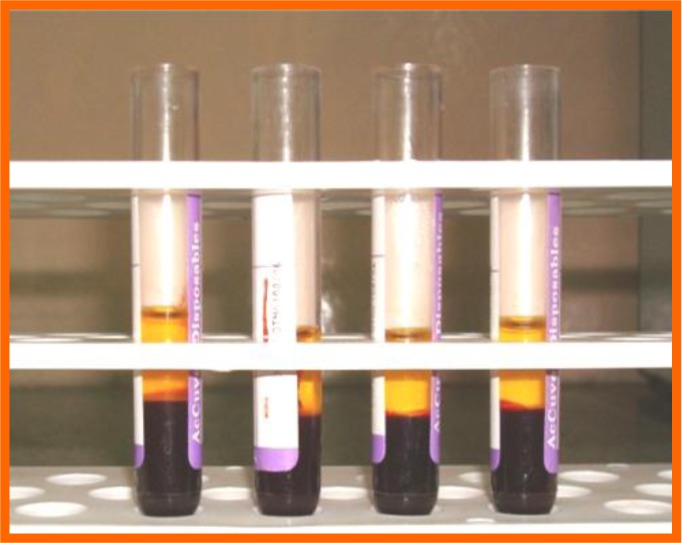
For 2^nd^ centrifuge.

**Fig. (7):**
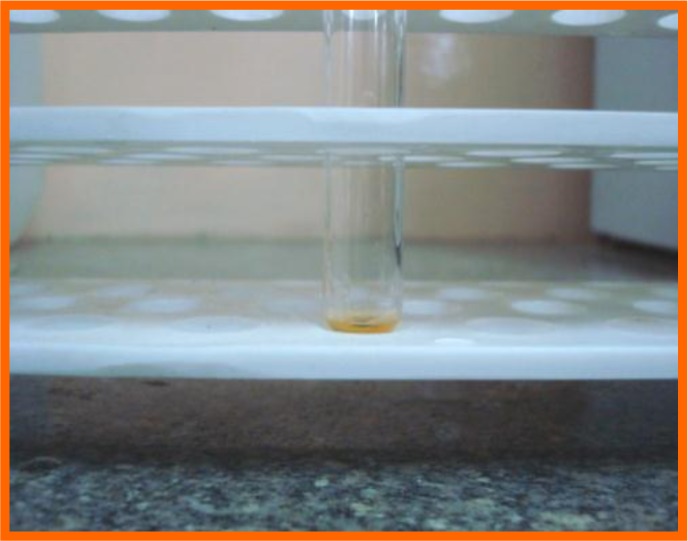
0.5 ml platelet rich plasma.

**Fig. (8):**
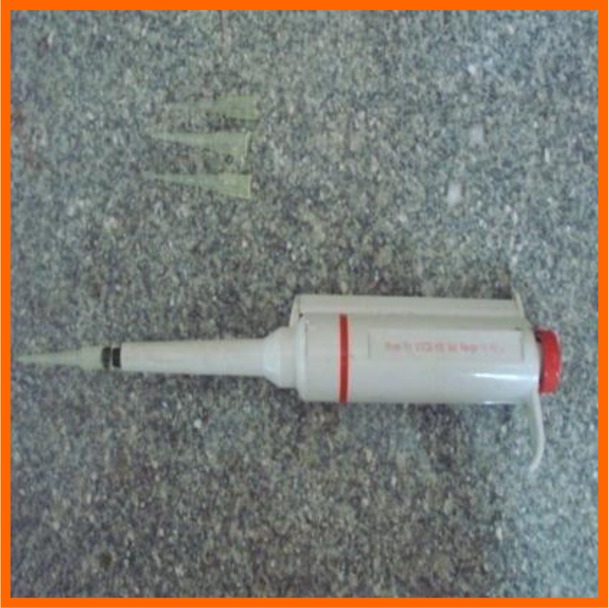
Pipette and micro tip.

PRP was prepared according to the procedure described by Kazuhiro Okuda *et al*. [16]. One hour prior to the periodontal surgery 8-10 ml of whole blood was drawn from the patient’s antecubital vein. Blood was collected in a vaccutainer coated with an EDTA. The tubes were inverted several times to ensure the mixing of blood and anticoagulant. The sample tube is then spun in a standard centrifuge (Remi 8x15 ml Capacity Laboratory Centrifuge R-303) for 10 minutes at 2100 rpm to separate PRP and platelet poor plasma (PPP) from the red blood cell fraction. The PPP was discarded, leaving just about 1ml of PPP present above the buffy coat. The 1ml of PPP, the whole of buffy coat and 1ml of red blood cell fraction rich in newly synthesized platelets was pipetted out and transferred to another test tube without an anticoagulant. The test tubes were centrifuged at 3500 rpm for 15 minutes, to separate PRP and PPP. The RPM was standardized according to the manual provided by the company having 5 speeds, starting with 700 rpm and so on with maximum speed of 3500 RPM (5x700 RPM). The PRP was then drawn into a syringe and expressed into a sterile container (Figs. **4**-**8**).

### 3.8 Stent Fabrication

A sterile perforated stock metal impression tray for each patient was selected. The impressions were made using an irreversible hydrocolloid material (alginate) following which casts were poured utilizing dental stone. Occlusal stent using pink polymerizing resin were then fabricated in the area of interest.

### 3.9 Pre-Surgical Clinical Measurements

The PI, GI, PGM, PPD, CAL were recorded.

#### 3.9.1 Surgical Procedure

**Fig. (9):**
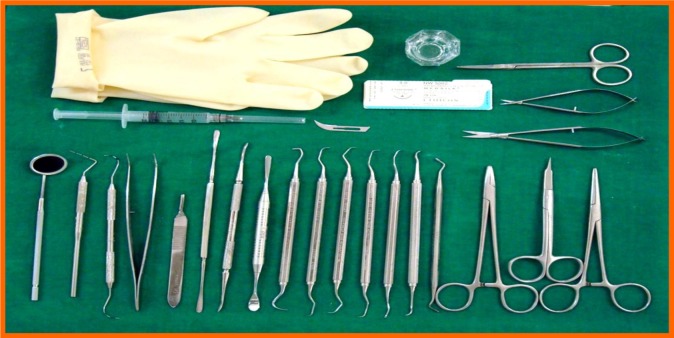
Surgical armamentarium.

Surgical area was anesthetized using local anesthetic (2% lignocaine with adrenaline 1:80000) and intracrevicular incisions were made and full thickness flap were elevated, to retain as much soft tissue as possible in order to obtain primary closure. The periodontal surgical procedure fully exposed the intrabony defects and meticulous defect debridement and root planing were carried out to remove subgingival plaque, calculus, diseased granulation tissue and pocket epithelium. The surgical sites were irrigated with sterile saline and care was taken to keep the area free of saliva and flaps were engaged using 3-0 silk suture material utilizing an interdental direct suturing technique (Fig. **9**).

Immediately before application the PRP was activated by clot initiator 10% calcium chloride and whole blood from the defect, within a few seconds the PRP preparation assumed a sticky gel consistency that will be relatively easy to apply to the surgical defects. The coagulated PRP and bone graft was placed upto the vertical height of the corresponding adjacent bone level using syringe and cumine scaler respectively. Flaps were repositioned to the pre-surgical level and the previously placed loose sutures at the sites were approximated and stabilised to achieve a primary closure (Figs. **10**, **11**).

### 3.10 Post Treatment Assessments

PI, GI, PGM, PPD, CAL were recorded at three and six months and radiographic assessments (Fig. **12A**) were done after 6 months postoperatively and oral hygiene instructions were reinforced.

### 3.11 Statistical Analysis

The data at baseline, 3 months and 6 months after surgery was first collected. Following this, the data was subjected to statistical analysis using:

-ANOVA
-Paired t-Test

All data were expressed as mean (SD). Statistical analysis was performed using a commercial SPSS version 18. One way analysis of variance was applied to examine the difference among the 2 groups. Clinical and radiographic parameters were subjected to student t-test and the “t” and *“p”* values were obtained with appropriate levels of significance.

## 4 RESULTS

### 4.1 Plaque Index

#### 4.1.1 Comparisons Within the Groups

**Graph. (1):**
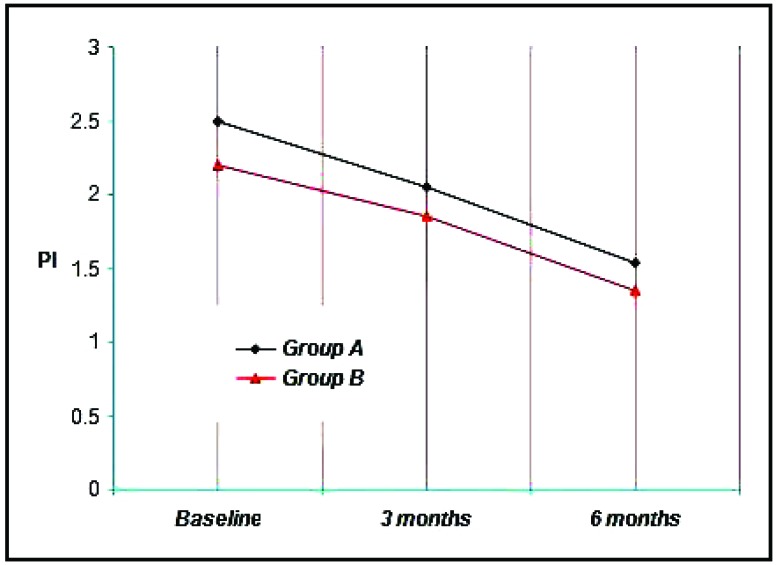
Showing the mean plaque index among the study groups at various time intervals.

Group A & B showed a mean plaque index of 2.50, 2.20 respectively at Baseline, 2.05, 1.85 at three months and 1.53, 1.35 at the end of six months. Thus a mean reduction of 0.45, 0.35 at three months and 0.97, 0.85 at six months was achieved which was statistically significant at (*p*<0.001) (Graph **1**).

#### 4.1.2 Comparisons Within the Groups Baseline Plaque Score

The mean plaque index at baseline for GROUP A was 2.50 ± 2.05, GROUP B was 2.20 ± 0.61. It is not found to be statistically significant.

##### 4.1.2.1 Baseline Plaque Score

The mean plaque index for Group A was 2.05 ± 0.55, Group B was 1.85 ± 0.69. It is not found to be statistically significant.

##### 4.1.2.2 Plaque Score at the End of 6 Months

The mean plaque index at 6 months for Group A was 1.53 ± 0.62, Group B was 1.35 ± 0.68. It is not found to be statistically significant.

When comparison was done between the 2 groups it was not found to bestatistically significant (*p*<0.05). In each group there was definitive reduction in plaque score over a period of time.

### 4.2 Gingival Index

#### 4.2.1 Comparisons Within the Groups

**Graph. (2):**
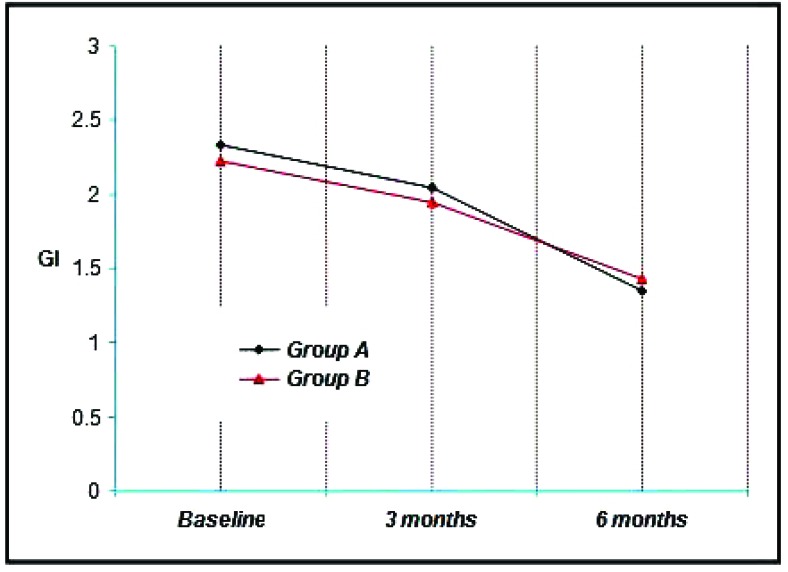
Showing the mean gingival index among the study groups at various time intervals.

Group A & B showed a mean gingival index of 2.33 & 2.23 respectively at Baseline, 2.05, 1.95 at three months and 1.35, 1.43 at the end of six months. Thus a mean reduction of 0.28, 0.28 at three months and 0.98, 0.80 at six months was achieved which was statistically significant at (*p*<0.001) (Graph **2**).

#### 4.2.2 Comparisons Between the Groups

##### 4.2.2.1 Baseline Gingival Score

The mean gingival index at baseline for Group A was 2.33 ± 0.65, Group B was 2.23 ± 0.48. It is not found to be statistically significant.

##### 4.2.2.2 Gingival Score at the End of 3 Months

The mean gingival index for Group A was 2.05 ± 0.67 and Group B was 1.95 ± 0 56 at 3 months. It is not found to be statistically significant.

##### 4.2.2.3 Gingival Score at the End of 6 Months

The mean gingival index at 6 months for Group A was 1.35 ± 0.58, Group B was 1.43 ± 0.54. It is not found to be statistically significant.

When comparison was done between the 2 groups it was not found to bestatistically significant (*p*<0.05), but each group showed improvement in gingival condition over a period of time.

### 4.3 Position of Gingival Margin (PGM)

#### 4.3.1 Comparisons Within the Groups

**Graph. (3):**
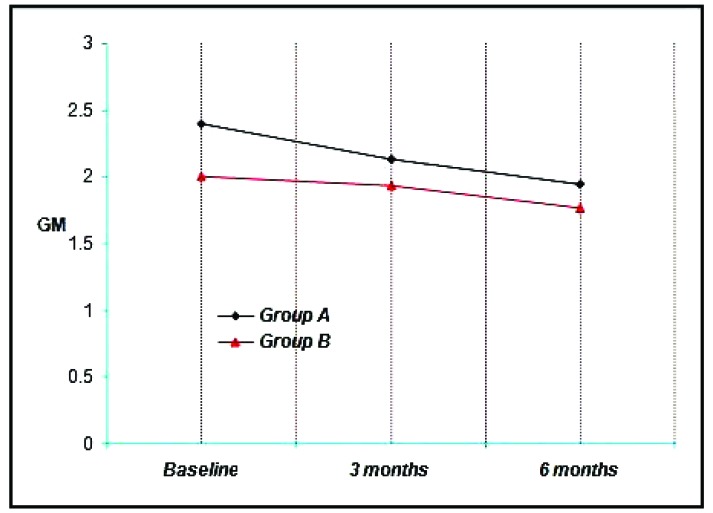
Showing the mean position of gingival margin among the study groups at various time intervals.

Group A & B showed a mean PGM of 2.40 & 2.00 respectively at Baseline, 2.13, 1.93 at three months and 1.94, 1.77 at the end of six months. Thus a mean reduction of 0.27, 0.07 at three months and 0.46, 0.23 at six months was achieved which was not statistically significant. (*p*<0.001) (Graph **3**).

#### 4.3.2 Comparisons Between the Groups

##### 4.3.2.1 Baseline Values

The mean PGM at baseline for Group A was 2.40 ± 0.57, Group B was 2.00 ± 0.47. It is not found to be statistically significant.

##### 4.3.2.2 Values at the End of 3 Months

The mean PGM for Group A was 2.13 ± 0.49, Group B was 1.93 ± 0.44. It is not found to be statistically significant.

##### 4.3.2.3 Values at the End of 6 Months

The mean PGM at 6 months for Group A was 1.94 ± 0.47, Group B was 1.77 ± 0.44. It is not found to be statistically significant.

When comparison was done between the 2 groups it was not found to bestatistically significant (*p*<0.05). In all the groups the procedures showed some degree of gingival shrinkage at the end of study period.

### 4.4 Probing Pocket Depth (PPD)

#### 4.4.1 Comparisons Within the Groups

**Graph. (4):**
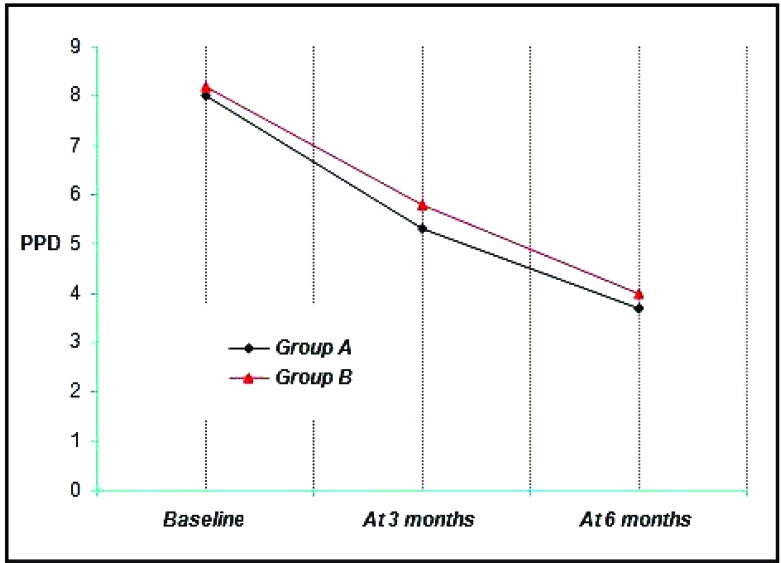
Showing the mean probing pocket depth among the study groups at various time intervals.

Group A & B showed a mean probing pocket depth (PPD) of 8 mm and 8.20 mm respectively at Baseline, 5.3 mm, 5.80 mm at three months and 3.7 mm, 4.00 mm at the end of six months. Thus a mean reduction of 2.70 mm, 2.40 mm at three months and 4.3 mm, 4.2 mm at six months was achieved which was statistically significant at (*p*<0.001) (Graph **4**).

#### 4.4.2 Comparisons Between the Groups

##### 4.4.2.1 Baseline Values

The probing pocket depth values for the sites ranged from 6 to 10 mm with a mean for Group A and 8.2 ± 0.91 mm.

##### 4.4.2.2 Values at the End of 3 Months

At 3 months, probing pocket depth values for the four groups ranged from 3 mm to 7 mm with a mean of 5.3 ± 1.16 mm for Group A, 5.80 ± 0.63 mm for the Group B (Graph **4**). It is not found to be statistically significant.

##### 4.4.2.3 Values at the End of 6 Months

At 6 months, probing pocket depth values for the four groups ranged from 2 mm to 5 mm with a mean of 3.70 ± 1.06 mm for Group A, 4.00 ± 1.05 mm for the Group B (Graph **4**). It is not found to be statistically significant.

When comparison was done between the 2 groups it was not found to be statistically significant (*p*<0.05). All the groups resulted in significant reductions in probing pocket depth (Graph **4**).

### 4.5 Clinical Attachment Level (CAL)

#### 4.5.1 Comparisons Within the Groups

**Graph. (5):**
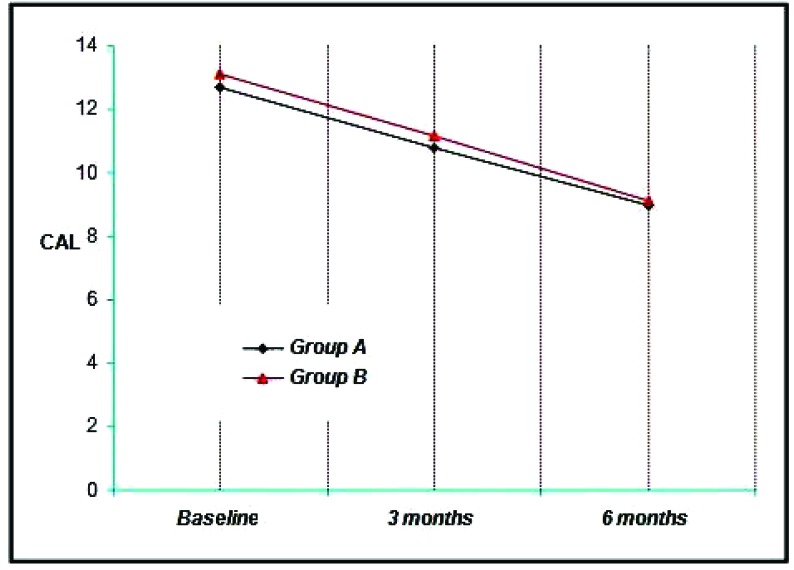
Showing the mean clinical attachment level among the study groups at various time intervals.

The Group A showed a mean clinical attachment level (CAL) of 12.70 mm at Baseline, 10.80 mm at 3 months and 9.00 mm at the end of six months. Thus a mean reduction of 1.90 mm at three months and 3.70 mm at six months was achieved which was statistically significant at (*p*<0.001) (Graph **5**).

The mean CAL in the Group B was 13.10 mm at baseline, 11.14 mm at three months and 9.10 mm at the end of 6 months, showing a mean reduction of 1.96 mm at three months and 4.00 mm at six months, which was also statistically significant. (*p*<0.001) (Graph Grp5).

#### 4.5.2 Comparisons Between the Groups

##### 4.5.2.1 Baseline Values

The Clinical attachment level values for the sites ranged from 8 to 14 mm with a mean of 12.70 ± 1.49 mm for Group A and 13.10 ± 0.74 mm for the Group B (Graph **5**). It is found to be statistically significant.

##### 4.5.2.2 Values at the End of 3 Months

At 3 months, clinical attachment level values for the four groups ranged from 7 mm to 14 mm with a mean of 10.80 ± 1.39 mm for Group A, 11.14 ± 0.97 mm for the Group B (Graph **5**). It is found to be statistically significant.

##### 4.5.2.3 Values at the End of 6 Months

At 6 months, clinical attachment level values for the four groups ranged from 8 mm to 13 mm with a mean of 9.00 ± 1.45 mm for Group A, 9.10 ± 0.9 mm for the Group B (Graph **5**). It is found to be statistically significant.

**Graph. (6):**
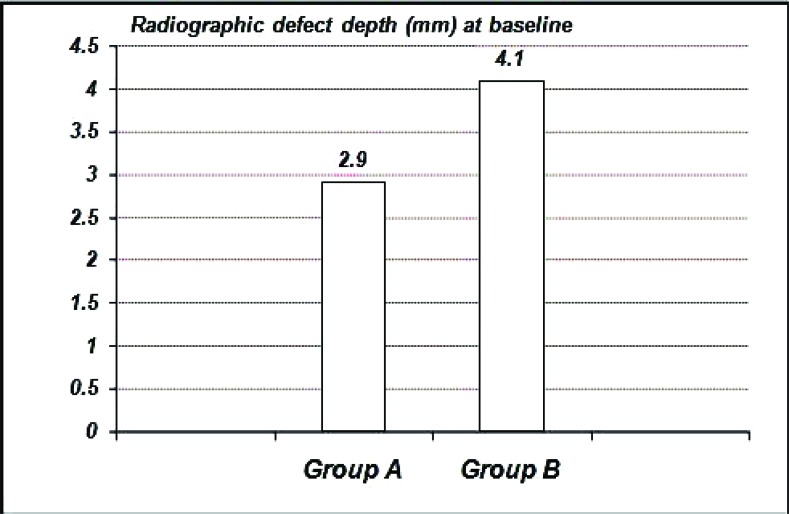
Showing the mean radiographic defect depth among the study groups at base line.

All the groups resulted in significant reductions in CAL (Graph **5**, **6**).

### 4.6 Radiographic Bone Level (RBL)

**Graph. (7):**
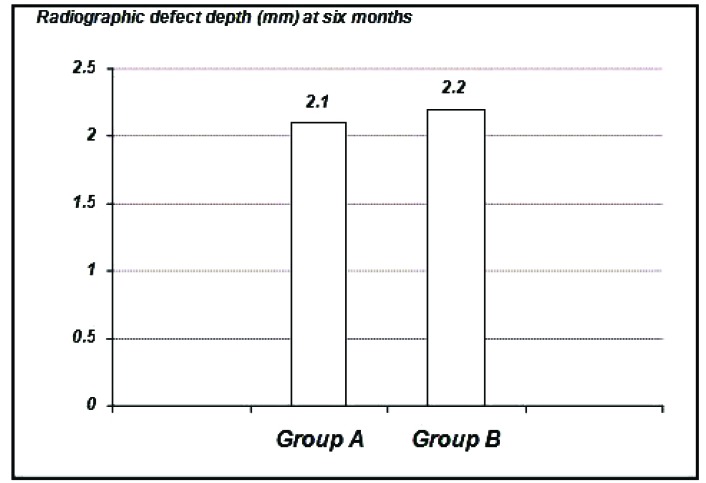
Showing the mean radiographic defect depth among the study groups at six months.

**Graph. (8):**
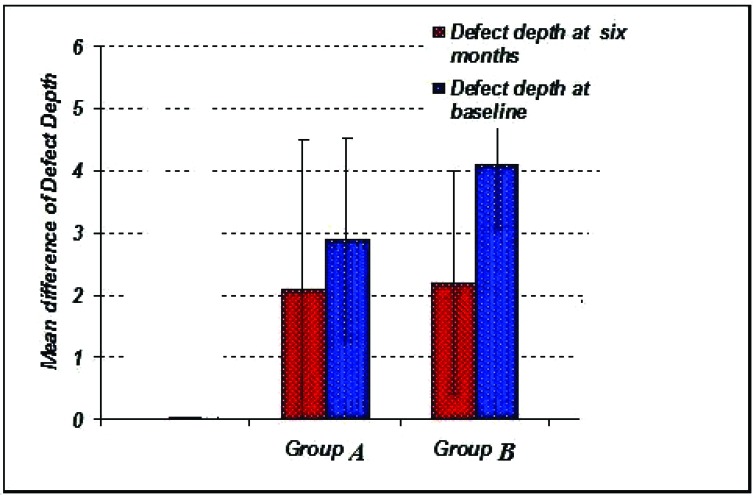
Showing the mean radiographic difference in defect depth among the study groups.

The radiographic defect depth values at baseline for Group A showed 2.90 ± 1.45 mm, Group B showed 4.10 ± 1.75 mm. (Graph **6**-**8**).

The radiographic defect depth values at six months: For Group Ashowed a mean defect depth of 2.10 ± 1.25 mm, Group B showed 2.20 ± 1.35 mm. (Graph **6**-**8**).

**Graph. (9):**
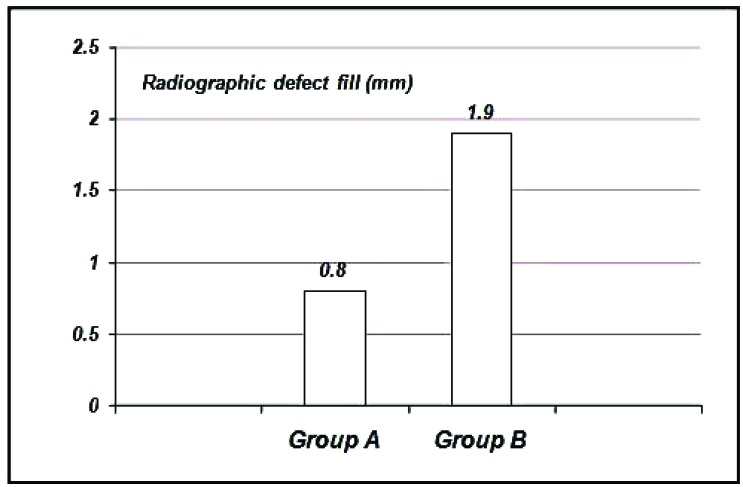
Showing the mean radiographic defect fill at six months among the study groups.

The radiographic defect fill values: For Group A showed a mean defect fill of 0.80 ± 0.79 mm, Group B showed 1.90 ± 0.84 mm. (Graph **9**).

**Graph. (10):**
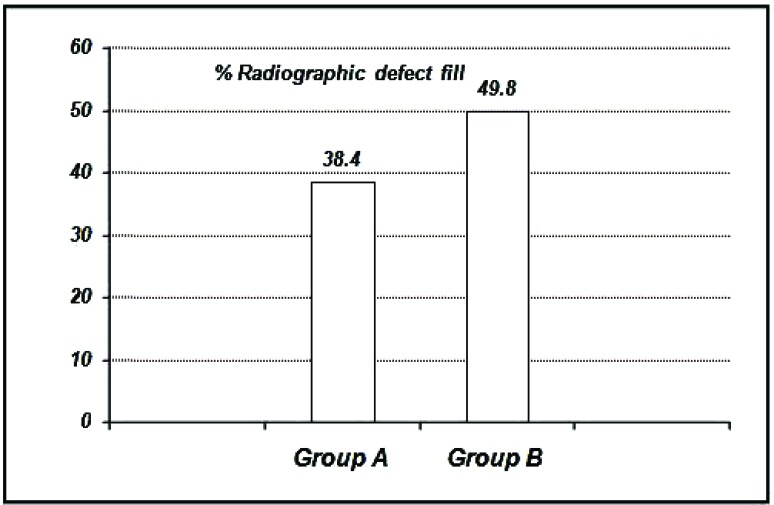
Showing the mean percentage of radiographic defect fill among the study groups at six months.

**Graph. (11):**
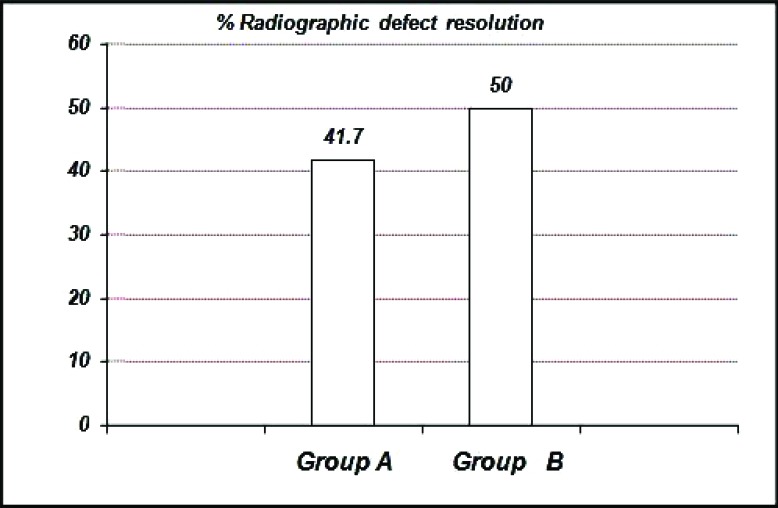
Showing the mean percentage of radiographic defect resolution among the study groups at six months.

The percentage of radiographic defect fill inGroupA is 38.40 ± 0.33%, Group B is 49.80 ±0.34% (Graph **10**, **11**).

The percentage of radiographic resolution of the defect in Group A is 41.70 ± 0.32%, Group B is 50.00 ± 0.27% (Graph **11**).

Thus, although the sites treated with bonegraft showed higher percentage ofradiographic defect fill when compared to PRP alone, no statistically significant differencein the percentage defect fill was found between the twogroups when measured at 6 months (*p*>0.05).

## 5 DISCUSSION

The ultimate goal of periodontal therapy is the creation of an environmental that is conducive to maintain patient dentition in a state of optimum health, comfort and function [17]. The deep intraosseous periodontal defects presents a major challenge in achieving the goal as it increases the risk of disease progression and recurrence after systematic traditional periodontal therapy. Regenerative periodontal therapy aims to reform and reconstitute the supporting tissues of teeth which have been lost due to periodontal disease and trauma. Several regenerative therapeutic procedures have been developed for this purpose; have met with partial or marginal success [17]. These include root surface biomodification, use of various types of bone grafts, guided tissue regeneration and combination of the above.

The platelet rich plasma is an autologous volume of plasma with 4-5 fold increase in platelet concentration, is a proven source of growth factors like PDGF, TGF, IGF, VEGF, EGF, Platelet derived angiogenesis factor and Platelet factor IV. PRP has been used for tissue regeneration in combination with autogenousbonegrafts in maxillofacial surgery. It has been used in periodontal defects with various alloplasts, bovine and natural porous bone mineral along with barrier membrane. [15-17]. The positive impact of PRP on bone healing is attributed to angiogenic, proliferate and differentiating effect of PDGF and TGF present in high concentration. However, there is pausity of information about the clinical efficacy of PRP in repair and regeneration of periodontal defect. Hence, the present study was designed to evaluate the regenerative potential of PRP and bone graft in periodontal osseous defects.

Present study was a clinico-radiographic study on evaluation of effect of PRP & bone graft in intra-bony defects that revealed a significant reduction in gingival index, plaque index, probing pocket depth and gain in clinical attachment level at various time intervals within both the groups. Radiographic evaluation revealed statistically significant defect fill (*p*<0.001) at the end of the study within both the groups. However, there was a difference seen statistically in Group B when compared to that of Group A, radiographically.

The plaque and gingival index were assessed at baseline, three months and six months in order to monitor patient’s oral hygiene and its effects on the soft tissue as this goes a long way in achieving the desired objective. The results of our investigation showed a statistically significant decrease in the plaque index from baseline to three months and at the end of six months in Group A as well as Group B, which is in accordance with a studywhere inthey concluded that ABBM + GTR (anorganic bovine bone mineral + guided tissue regeneration) with a non-resorbable barrier, with or without the addition of PRP, showed optimal clinical results [18].

The changes in PPD reflect the cumulative effect of the response of gingival tissue to the treatment by way of gingival recession and clinical attachment gain. PPD indicates the volume of subgingival area, which harbours the pathogenic microbiota and favors disease activity. Change in CAL following regenerative therapy is the single most commonly used outcome measure in regenerative therapy. This is based on reported correlation between gain in CAL and gain in bone height by various clinical studies [19]. The results of our study showed a CAL gain both in group A and B which was statistically significant. This is in accordance with the study where they concluded that both scaling and root planing alone and scaling and root planing combined with flap procedure are effective methods for the treatment of chronic periodontitis in terms of attachment level gain and reduction ingingival inflammation [20].

New bone formation is frequently used as an important outcome variable in controlled clinical trials for regenerative procedures. Radiographic monitoring of alveolar bone changes following regenerative procedure is a non-invasive painless alternative to direct bone measurement which is done by re-entry procedures. Radiographic variables assessed in our study were extent of defect fill and defect resolution using image analysis software (auto-cad analysis). As the radiographic changes cannot be appreciated at three months interval they were recorded at 6 months interval.

The results of our study shows the defect fill in group B was 1.90 ± 0.84 mm suggesting that HA+TCP bonegraft is superior to PRP alone in bringing about bone formation. Similar findings have been reported earlier also by different investigators [21]. The difference between bone fill and bone resolution is because of the alteration of crestal bone height observed in the groups. Crestal bone resorption is a characteristic feature after the flap procedure. However,one study by showed that alloplasts in combination with PRP have reported insignificant amount of resorption though not total prevention or regeneration of crestal bone [22]. whereas another study has reported crestal bone resorption with bovine porous bone mineral and PRP in treatment of human intrabony defects supporting our findings [21].

In a meta analysis done to evaluate the effectiveness of PRP for periodontal regeneration in the treatment of intrabony defects, it was concluded that PRP might offer some beneficial effects on clinical and radiographic outcomes for regeneration of periodontal intrabony defects [23]. The results of the present study is in accordance with the same, where there was decrease in the PPD and initiation of bone fill when the intra bony defects were treated using PRP. A systematic review and meta analysis that was conducted to evaluate the effect of platelet-rich plasma on clinical outcomes of the surgical treatment of periodontal intrabony defects suggested that PRP may be beneficial as an adjunct for the treatment of periodontal intra bony defects [24]. According to the results of our present study, although both the groups showed a significant difference in the clinical parameters but the use of bone grafts showed a better result in terms of bone formation. Hence it can be suggested that PRP when used along with bone graft might present with superior results.

In a systematic review it was concluded that there was significant increase in bone formation on radiographs in the PRP group and in vivo studies evaluated, suggested that PRP confers several beneficial effects on animal long-bone models [25]. It was determined that PRP can be used as a biologic adjunct in long-bone models. In the current study statistical significant difference was seen in clinical parameters and radiographic evaluation which is in accordance with the results of the systematic review.

Within the limits of present investigation it could be concluded that bone graft has beneficiary activity when compared to PRP.

## CONCLUSION

The 2 treatment modalities (Group A & B) showed favourableclinical results. Bonegrafts showed better results in comparison with PRP. It is yet not clearly known whether PRP alone or with combination with bonegraft would significantly enhance the outcome of regenerative periodontal therapy. PRP application is of great interest to the researcher as well as clinician, holds promise and needs further exploration.

## Figures and Tables

**Fig. (10A):**
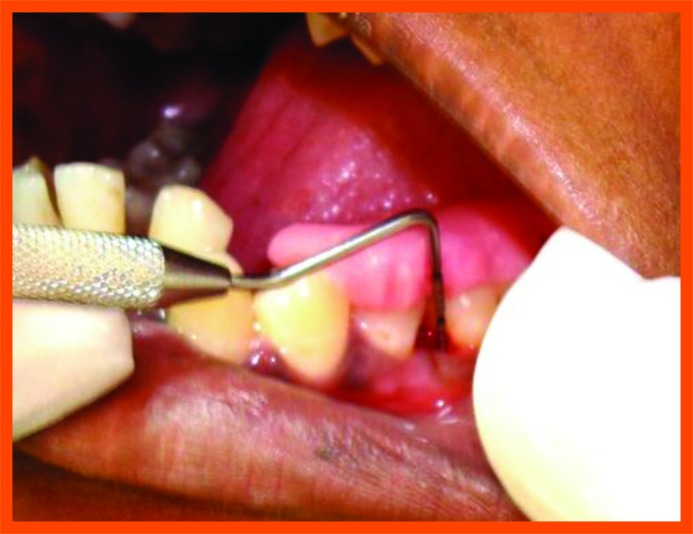
Probing with occlusal stent.

**Fig. (10B):**
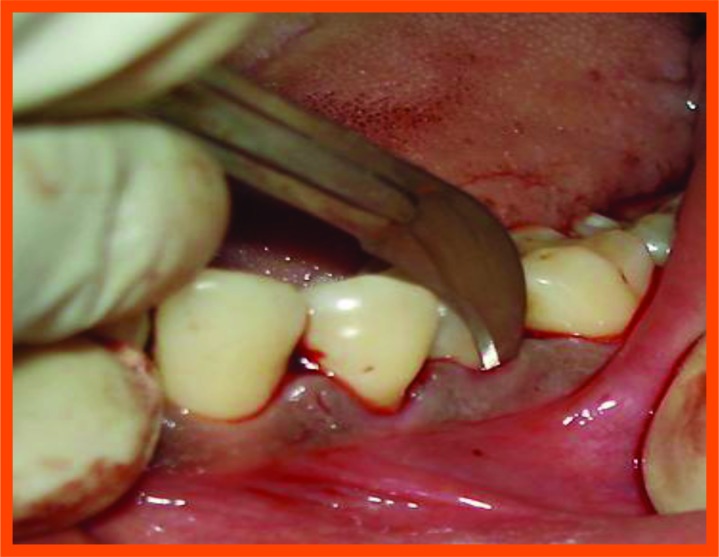
Incision placed.

**Fig. (10C):**
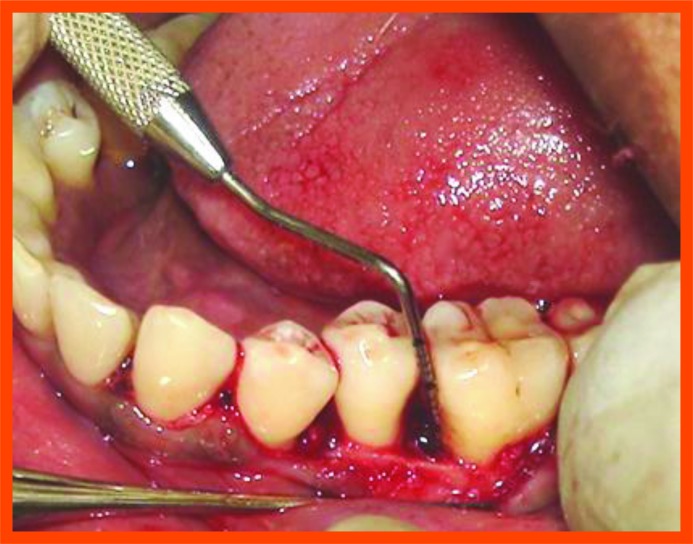
Reflection and debridement.

**Fig. (10D):**
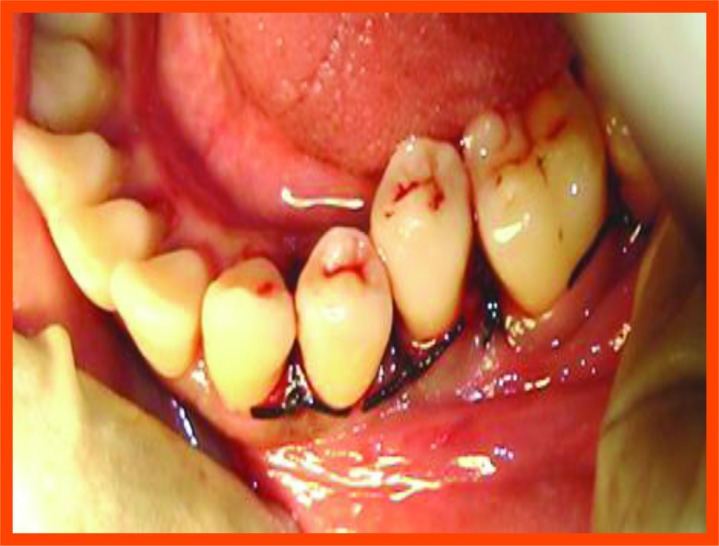
PRP placed and suture placement.

**Fig. (10E):**
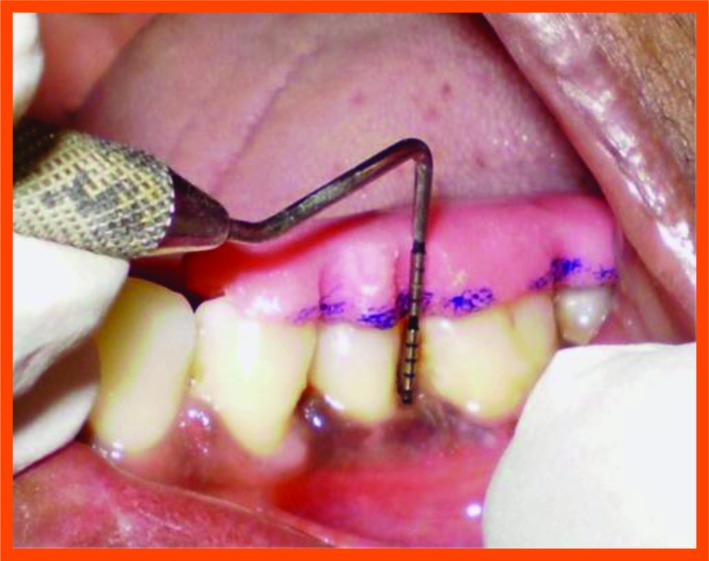
Probing after 6 months.

**Fig. (11A):**
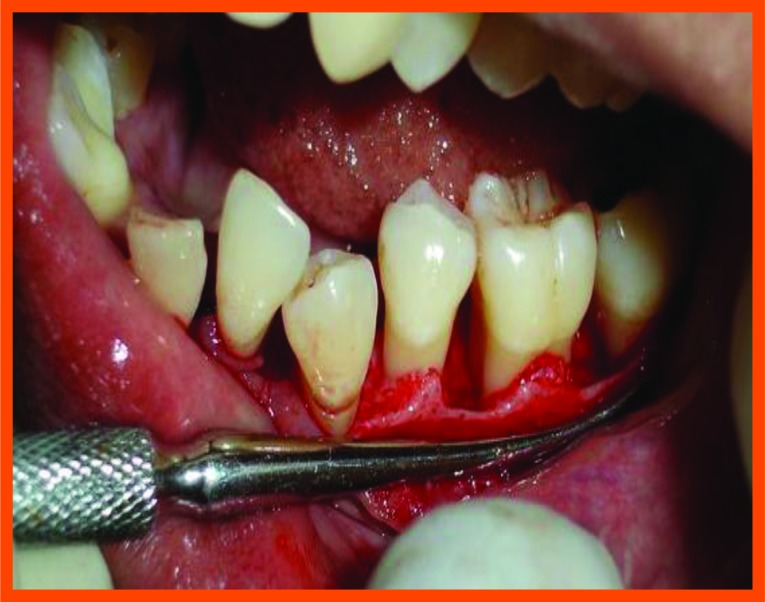
Reflection and debridement done.

**Fig. (11B):**
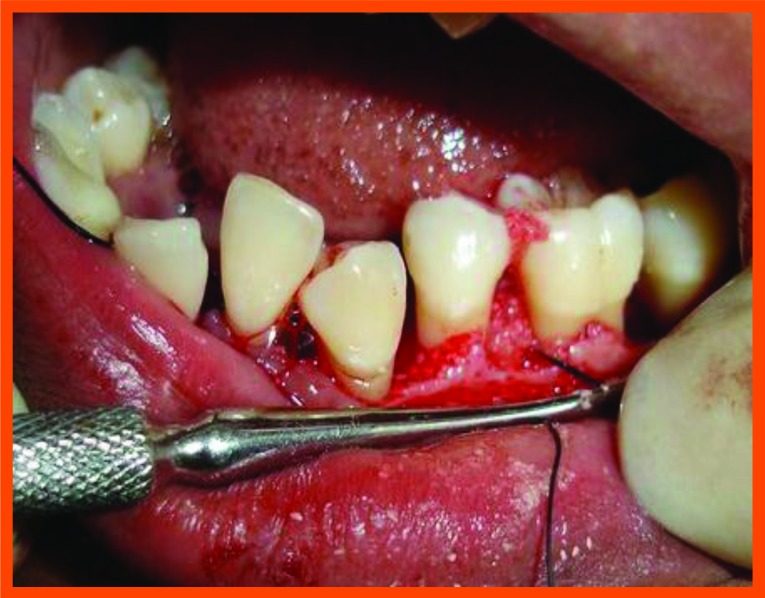
Bonegraft placed.

**Fig. (11C):**
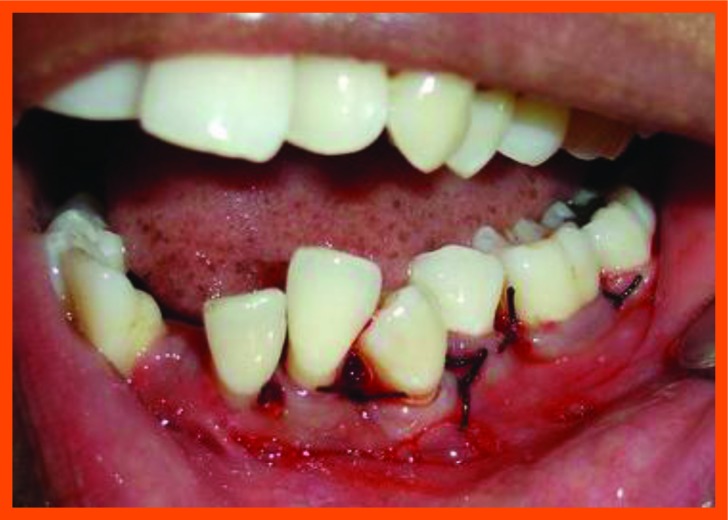
Suture placement.

**Fig. (12A):**
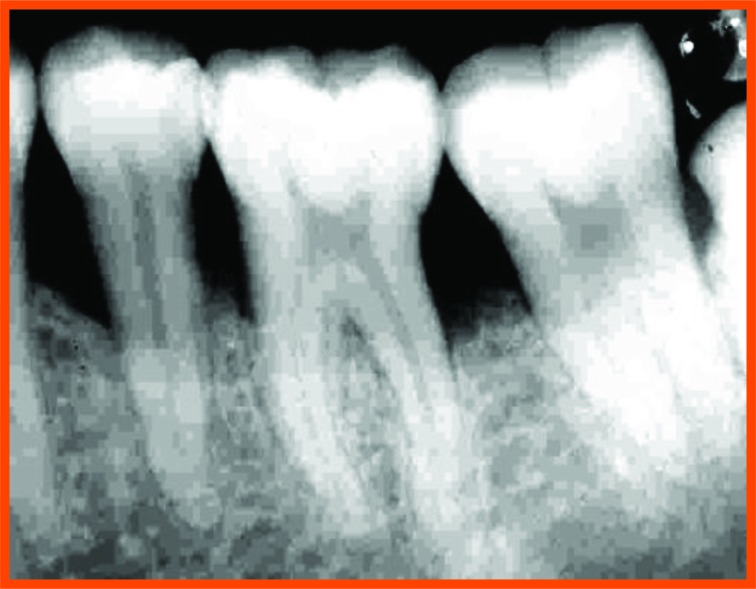
PRP group (group a): Pre operative distal aspect of 36.

**Fig. (12B):**
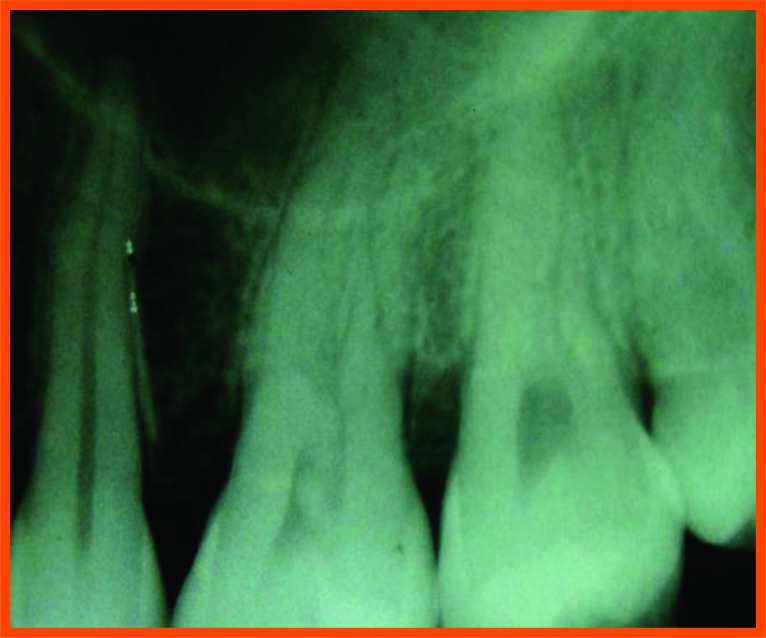
Pre operative mesial aspect of 26.

**Fig. (12C):**
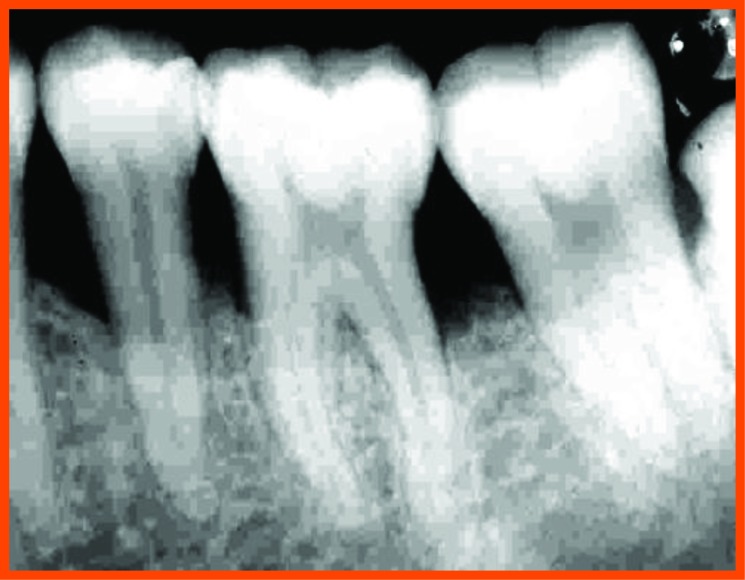
6 Month post operative distal aspect of 36 distal.

**Fig. (12D):**
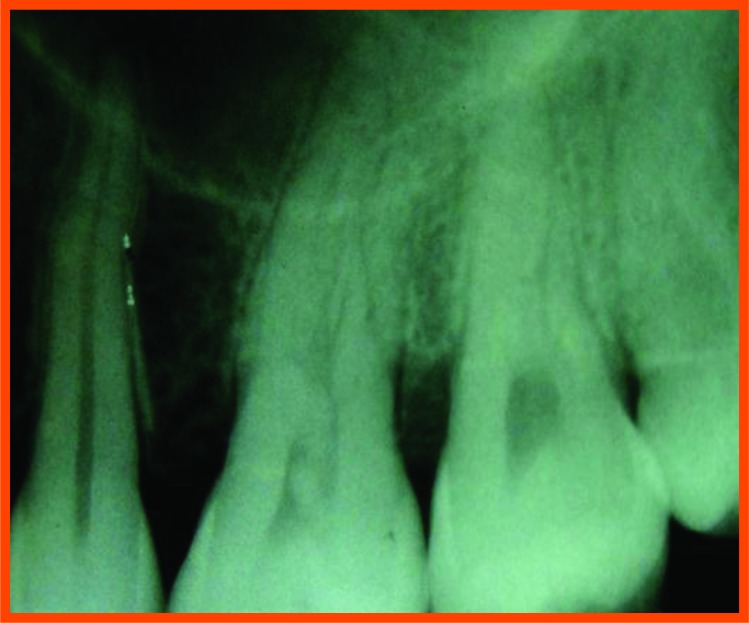
Post operative- 6 month mesial aspect of 26.

**Fig. (12E):**
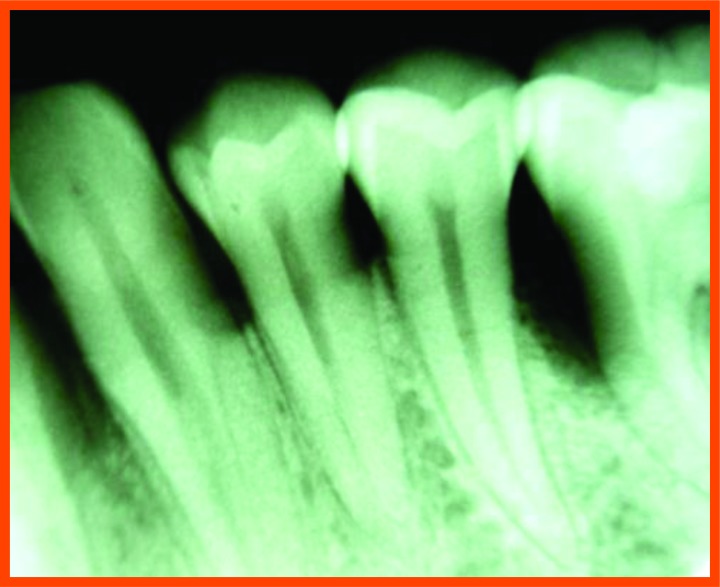
Pre-operative mesial aspect of 46.

**Fig. (12F):**
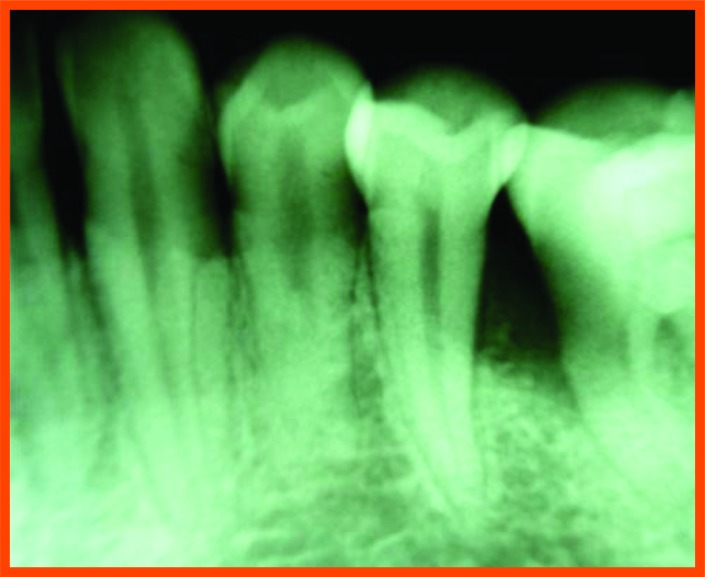
Post-operative -6 month mesial aspect of 46.

**Fig. (12G):**
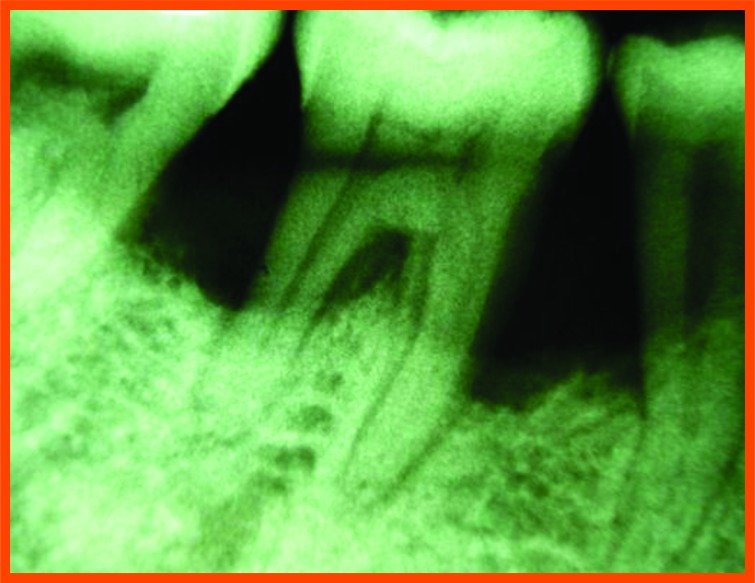
Pre-operative mesial aspect of 36.

**Fig. (12H):**
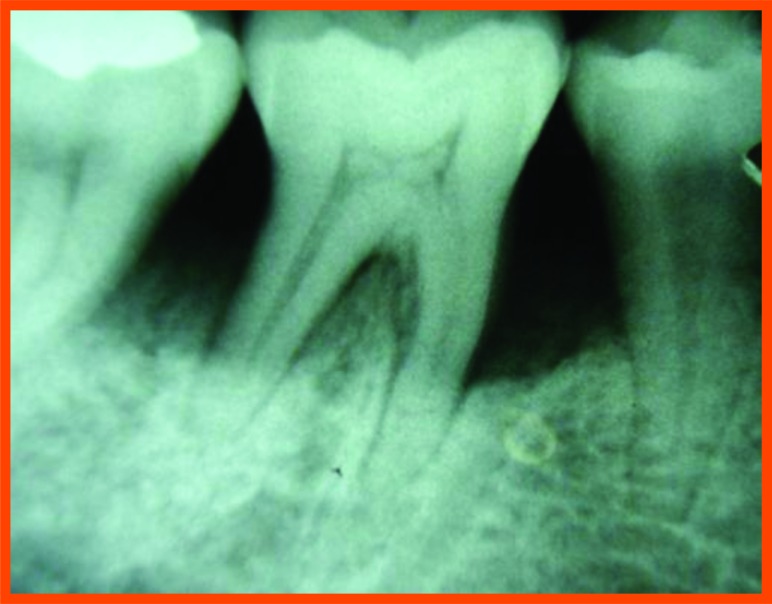
Post-operative -6 month mesial aspect of 36.
